# Influenza A(H6N1) Virus in Dogs, Taiwan

**DOI:** 10.3201/eid2112.141229

**Published:** 2015-12

**Authors:** Hui-Ting Lin, Ching-Ho Wang, Ling-Ling Chueh, Bi-Ling Su, Lih-Chiann Wang

**Affiliations:** National Taiwan University, Taipei, Taiwan

**Keywords:** influenza A(H6N1) virus, viruses, influenza, avian influenza virus, dogs, H6N1 subtype, Taiwan

## Abstract

We determined the prevalence of influenza A virus in dogs in Taiwan and isolated A/canine/Taiwan/E01/2014. Molecular analysis indicated that this isolate was closely related to influenza A(H6N1) viruses circulating in Taiwan and harbored the E627K substitution in the polymerase basic 2 protein, which indicated its ability to replicate in mammalian species.

Infections with influenza viruses are rare in dogs. However, interspecies transmission of an equine influenza A(H3N8) virus to dogs was identified during a respiratory disease outbreak in Florida, USA, in 2004 (*1*). Influenza A(H6N1) virus is the most common naturally occurring avian influenza virus in Taiwan (*2*). Therefore, to determine to the prevalence of influenza A virus infection in dogs in Taiwan, we performed serologic analysis, 1-step reverse transcription PCR (RT-PCR) screening, and virus isolation.

## The Study

A total 474 serum specimens were collected in Taiwan during October 2012–October 2013. Two hundred eighty-one specimens were collected from household (owned) dogs at the National Taiwan University Veterinary Hospital in Taipei. The remaining 193 serum specimens were obtained from free-roaming dogs in rural areas.

All serum specimens were tested for antibodies against influenza A virus by using a species-independent blocking ELISA (Influenza A Virus Antibody Test Kit; Idexx, Westbrook, ME, USA). All antibody-positive serum specimens were further tested by using a hemagglutination inhibition (HI) assay. HI was determined according to procedures recommended by the World Organisation for Animal Health. Chicken erythrocytes (1%) were used. Serum samples were treated with receptor-destroying enzyme (Denka Seiken, Tokyo, Japan) before conducting the assay to destroy nonspecific inhibitors (*3*). A/chicken/Taiwan/2838V/2000 (H6N1) and A/chicken/Taiwan/1209/03 (H5N2) viruses were used as antigens.

Nasal swab specimens were collected from dogs with respiratory signs, such as nasal discharge, sneezing, coughing, at the National Taiwan University Veterinary Hospital during November 2012–February 2014. Specimens were suspended in viral transportation medium (Creative, Taipei, Taiwan), and RNA was extracted by using a commercial kit (Viral RNA Mini Kit; QIAGEN, Hilden, Germany) according to the manufacturer’s instructions. A 1-step RT-PCR was then performed by using the One-Step RT-PCR Kit (QIAGEN). A primer set (M52C/M253R) specific for a highly conserved region of matrix (M) gene was used for detection of influenza A virus nucleotides (*4*). The remaining nasal swab suspension solutions from dogs positive by RT-PCR were used for virus isolation from 10-day-old specific pathogen–free chicken eggs (Animal Health Research Institute, Taipei, Taiwan).

Phylogenetic trees were constructed with complete nucleotide sequences obtained from the Global Initiative on Sharing All Influenza Data (http://platform.gisaid.org/epi3/frontend#185d95) and GenBank. Multiple sequence alignments and phylogenetic analyses were performed by using MEGA6 software (*5*). Sequences were aligned by using the ClustalW method (http://www.genome.jp/tools/clustalw/). Trees were constructed by using the maximum-likelihood method and analysis with 1,000 bootstrap replications.

A total of 3/281 (1.1%) household dogs and 6/193 (3.1%) free-roaming dogs were positive by ELISA for influenza A virus. The HI assay showed that 1 of the 9 virus-positive dogs had antibodies against influenza A(H6N1) virus (titer = 20). This dog was from Yunlin, Taiwan, a rural area that is a major site for poultry production. No serum samples had antibodies against influenza A(H5N2) virus.

Nasal swab specimens from 4/185 (2.1%) dogs were positive by RT-PCR for influenza virus M gene. All 4 RT-PCR positive dogs had nasal discharges or coughing. Three of these dogs were <6 months of age and adopted from an animal shelter (n = 1) or rescued from the streets (n = 2). The fourth dog was a 15-year-old household pet.

Influenza A virus was isolated from a 4-month-old dog co-infected with canine distemper virus. The virus influenza isolate was designated A/canine/Taiwan/E01/2014 (GenBank accession nos. KM20333–KM203344). This dog was rescued from the streets and had severe purulent nasal discharge, cough, and fever. Chest radiographs showed a severe bilateral air bronchogram in the lung field. Serum specimens (collected on days 1, 7, 14, and 19 after hospitalization) from this dog were assessed by using ELISA and HI assay, but no seroconversion was observed.

Sequence homology of 8 influenza virus gene segments from A/canine/Taiwan/E01/2014 (H6N1) was compared with segments in the Global Initiative on Sharing All Influenza Data (Table 1). Hemagglutinin (HA) and neuraminidase (NA) genes of this virus had the highest nucleotide sequence similarity (99%) with A/chicken/Taiwan/1843/2012 (H6N1) and A/chicken/Taiwan/2084/2012 (H6N1), respectively. Polymerase basic 2 (PB2), PB1, nucleoprotein (NP), and nonstructural protein (NS) genes were closely related to those of H6N1 subtype virus isolates from chickens in Taiwan (similarity range 97%–99%). Polymerase acidic (PA) and M genes had the highest nucleotide sequence similarity (99%) to A/chicken/Taiwan/2593/2012 (H5N2). On the basis of HA and NA sequence analysis results, A/canine/Taiwan/E01/2014 was designated an H6N1 subtype influenza virus.

**Table 1:** Homology of nucleotide sequences of A/canine/Taiwan/E01/2014 (H6N1) influenza virus isolated from dogs in Taiwan compared with related sequences from the Global Initiative on Sharing All Influenza Data*†

Gene segment	Virus with highest identity	% Identity	Accession no.
PB2	A/chicken/Taiwan/1843/2012 (H6N1)	98	EPI510830
PB1	A/chicken/Taiwan/A2837/2013 (H6N1)	97	EPI459872
PA	A/chicken/Taiwan/2593/2012 (H5N2)	99	EPI510622
HA	A/chicken/Taiwan/1843/2012 (H6N1)	99	EPI519832
NP	A/chicken/Taiwan/67/2013 (H6N1)	98	EPI510875
NA	A/chicken/Taiwan/2084/2012 (H6N1)	99	EPI510837
M	A/chicken/Taiwan/2593/2012 (H5N2)	99	EPI510660
NS	A/chicken/Taiwan/67/2013 (H6N1)	97	EPI510878

*All viruses were from avian sources. PB, polymerase basic; PA, polymerase acidic; HA, hemagglutinin; NP, nucleoprotein; NA, neuraminidase; M, matrix; NS, nonstructural protein. †http://platform.gisaid.org/epi3/frontend#185d95.

Only 1 basic amino acid (PQIATR*G) was found at the HA cleavage site of A/canine/Taiwan/E01/2014. G228S substitution (H3 numbering) on the receptor binding site for HA was also observed for this virus, which indicated increased virus binding ability for the α2–6 sialic acid receptor (*6**,**7*). In NA, a 14-aa deletion in the NA stalk region was observed at aa positions 42–53 and 68–69, which is associated with virus circulation in domestic poultry. The amino acid H275Y substitution (oseltamivir resistance marker) in NA was not found in this virus. In the M2 protein, A/canine/Taiwan/E01/2014 had an S31N substitution, which suggested resistance to admantanes (*8**,**9*).

Other major signatures associated with replication ability in a mammalian host or pathogenicity were also observed, including E627K in the PB2 and the PDZ ligand domain at the C-terminal region of NS1 of this virus. Additional molecular comparisons with H6N1subtype virus (A/Taiwan/2/2013) isolated from humans (*7**,**10**,**11*) and from dogs experimentally infected H6N1 subtype virus (A/mallard/San-Jiang/275/2007) (*12*) were made (Table 2).

**Table 2:** Molecular characterization of A/canine/Taiwan/E01/2014 (H6N1) influenza virus and 2 other influenza viruses, Taiwan*

Gene, amino acid substitution	Virus			Function
	A/canine/Taiwan/E01/2014	A/Taiwan/2/2013	A/mallard/San-Jiang/275/2007	
PB2				
E627K	K	E	E	Replication ability in mammalian host
D701Q	D	D	D	Nuclear import
PB1-F2				
N66S	N	Truncated form	S	Induction of apoptosis
HA				
Cleavage site	Single basic amino acid (PQIATR†G)	Single basic amino acid (PQIATR†G)	Single basic amino acid (PQIETR†G)	HA cleavage
Q226L	Q	Q	Q	Increased virus binding ability of α2–6 sialic acid receptor
G228S	S	S	G	
NA				
H275Y	H	H	H	Oseltamivir resistance
41–52 and 68–69 deletions	Deleted	Deleted	Complete	Adaptation hallmark of waterfowl viruses to terrestrial poultry
M2				
S31N	N	N	S	Adamantane resistance
NS1				
D92E	D	D	D	Unknown
EPEV sequence (C-terminus)	EPEV	EPEV	ESEV	PDZ ligand domain

*PB, polymerase basic; HA, hemagglutinin; NA, neuraminidase; M, matrix; NS, nonstructural protein. †HA cleavage site.

Phylogenetic analysis of HA and NA gene segments indicated that A/canine/Taiwan/E01/2014 belongs to the H6N1 lineage that has been circulating in chickens in Taiwan since 1997 (Figure, panels A, B). Although the lineage of internal gene segments (PB2, PB1, PA, NP, M, and NS) is composed mainly of H6N1 subtype viruses isolated in Taiwan, some H5N2 subtype isolates in the H6N1 lineage were observed (Figure, panel C; Technical Appendix).

**Figure:**
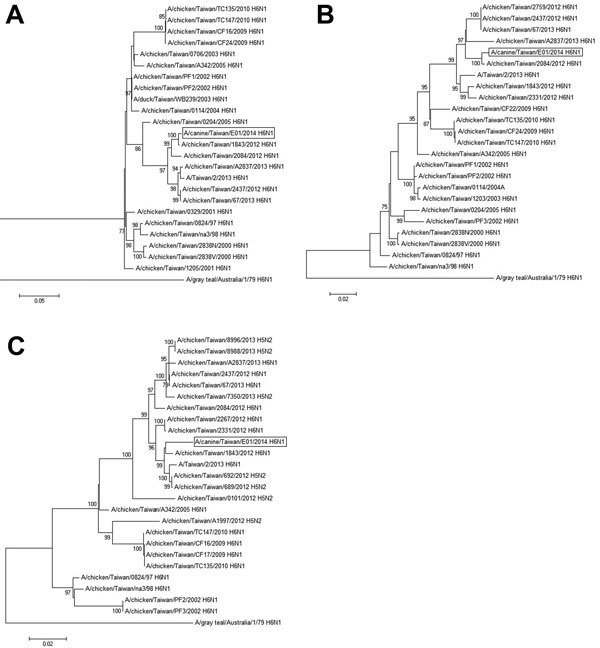
Phylogenetic relationship between influenza A(H6N1) virus A/canine/Taiwan/E01/2014 isolated from dogs in Taiwan (boxes) and other influenza A virus lineages. Maximum-likelihood analysis with bootstrap analysis was conducted with 1,000 replications. Only branches with bootstrap values >75% are indicated on phylogenetic trees. A) Hemagglutinin, B) neuraminidase, and C) polymerase basic 2 genes of A/canine/Taiwan/E01/2014 are clustered with H6N1 subtype strains isolated in Taiwan during 2012–2013 in Taiwan. Scale bars indicate nucleotide substitutions per site.

## Conclusions

Avian influenza A(H6N1) viruses have been widespread in chickens in Taiwan since 1972 (*13**–**15*). These viruses are clustered in a unique lineage that differs from viruses circulating in Hong Kong and southeastern China since 1997 (*13*). Unlike avian species, H6 subtype virus infections are rare in mammals.

In this study, 9 of 474 dog serum specimens were positive for influenza A virus by ELISA, and 4/185 (2.1%) dogs had RT-PCR−positive results for this virus. A/canine/Taiwan/E01/2014 was isolated from 1 dog that was co-infected with canine distemper virus. On the basis of molecular analysis of A/canine/Taiwan/E01/2014, HA, NA, PB1, PB2, NP, and NS genes showed high homology (>97% nucleotide identity) with avian H6N1 subtype virus isolates that are currently prevalent in Taiwan. PA and M genes of A/canine/Taiwan/E01/2014 showed 99% nucleotide identity with A/chicken/Taiwan/2593/2013 (H5N2).

Phylogenetic analysis showed that 8 eight virus genes were derived from H6N1 subtype viruses isolated in Taiwan. All 8 influenza virus genes found in the dog probably originated from avian sources. We speculate that a complete avian influenza virus had infected this dog. However, additional analysis is required to verify this hypothesis.

Technical Appendix Phylogenetic relationship between influenza A(H6N1) virus A/canine/Taiwan/E01/2014 isolated from dogs in Taiwan and other influenza A virus lineages.
