# Supplementary material for: Influenza A(H6N1) Virus in Dogs, Taiwan
Source: Emerg Infect Dis. 2015 Dec;21(12):2154–7. doi: 10.3201/eid2112.141229 (PMC4672433; doi:10.3201/eid2112.141229)
Supplement: Technical Appendix — Phylogenetic relationship between influenza A(H6N1) virus A/canine/Taiwan/E01/2014 isolated from dogs in Taiwan and other influenza A virus lineages. [file 14-1229-Techapp-s1.pdf]

Article DOI: http://dx.doi.org/10.3201/eid2112.141229

Influenza A(H6N1) Virus in Dogs, Taiwan

Technical Appendix

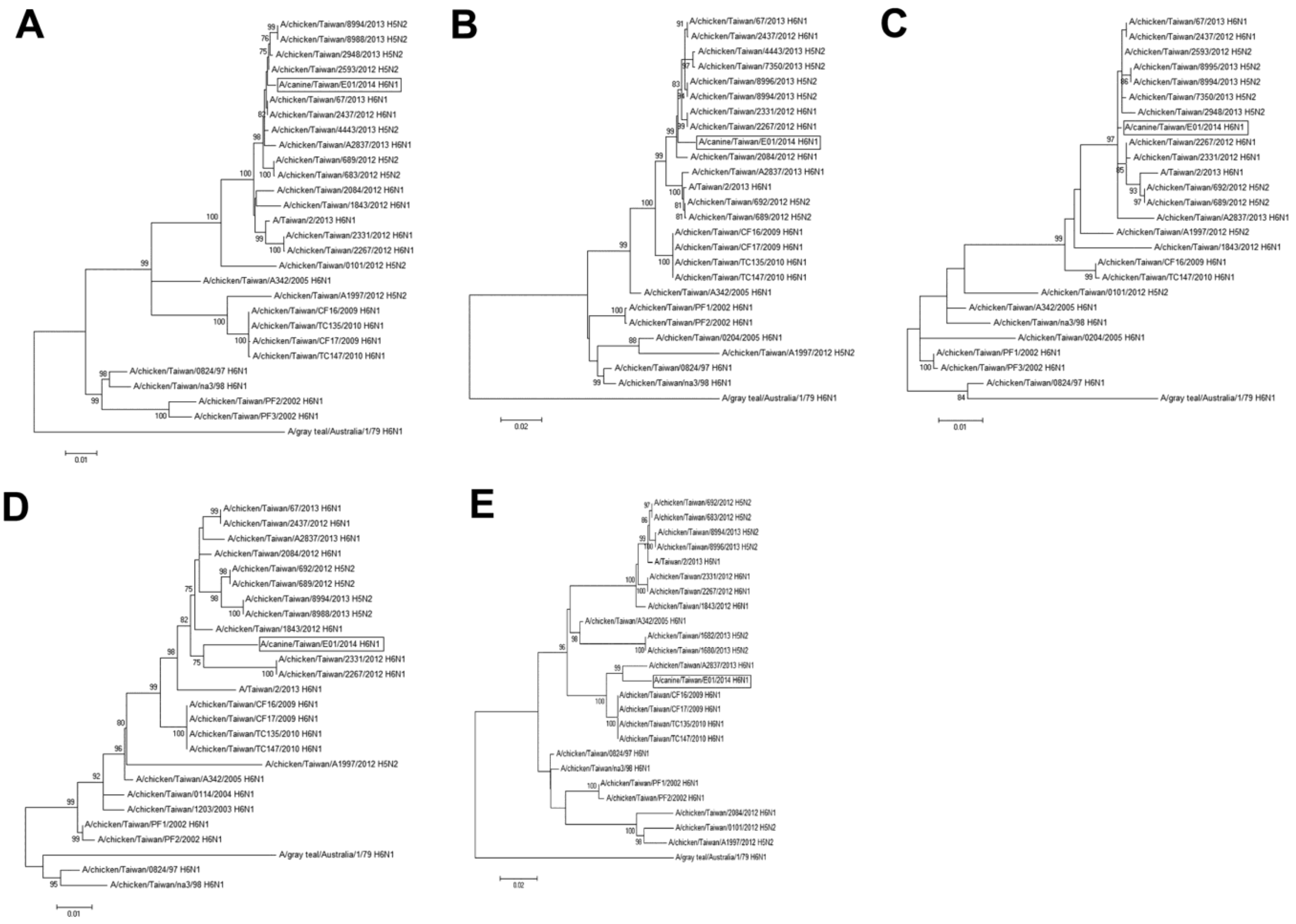

Technical Appendix Figure. Phylogenetic relationship between influenza A(H6N1) virus A/canine/Taiwan/E01/2014 isolated from dogs in Taiwan and other influenza A virus lineages. Boxes indicate strain isolated in this study. Maximum-likelihood with bootstrap analysis was conducted with 1,000 replications. Only branches with bootstrap values >75% are indicated on phylogenetic trees. A) Polymerase acidic, B) nucleoprotein, C) matrix, D) nonstructural protein, and E) polymerase basic 1 genes of A/canine/Taiwan/E01/2014 are clustered with H6N1 subtype trains isolated in Taiwan during 2012–2013 in Taiwan. Scale bars indicate nucleotide substitutions per site.
